# The Influence of Ventilation Measures on the Airborne Risk of Infection in Schools: A Scoping Review

**DOI:** 10.3390/ijerph20043746

**Published:** 2023-02-20

**Authors:** Sandra N. Jendrossek, Lukas A. Jurk, Kirsten Remmers, Yunus E. Cetin, Wolfgang Sunder, Martin Kriegel, Petra Gastmeier

**Affiliations:** 1 Institute of Hygiene and Environmental Medicine, Charité—Universitätsmedizin Berlin, Corporate Member of Freie Universität Berlin and Humboldt-Universität zu Berlin, 12203 Berlin, Germany; 2 Institute of Industrial Building and Construction Design, Technical University Carolo Wilhelmina, 38106 Braunschweig, Germany; 3 Hermann-Rietschel-Institut, Technical University of Berlin, 10623 Berlin, Germany

**Keywords:** school, ventilation, CO_2_ concentration, airborne transmission, SARS-CoV-2

## Abstract

Objectives: To review the risk of airborne infections in schools and evaluate the effect of intervention measures reported in field studies. Background: Schools are part of a country’s critical infrastructure. Good infection prevention measures are essential for reducing the risk of infection in schools as much as possible, since these are places where many individuals spend a great deal of time together every weekday in a small area where airborne pathogens can spread quickly. Appropriate ventilation can reduce the indoor concentration of airborne pathogens and reduce the risk of infection. Methods: A systematic search of the literature was conducted in the databases Embase, MEDLINE, and ScienceDirect using keywords such as school, classroom, ventilation, carbon dioxide (CO_2_) concentration, SARS-CoV-2, and airborne transmission. The primary endpoint of the studies selected was the risk of airborne infection or CO_2_ concentration as a surrogate parameter. Studies were grouped according to the study type. Results: We identified 30 studies that met the inclusion criteria, six of them intervention studies. When specific ventilation strategies were lacking in schools being investigated, CO_2_ concentrations were often above the recommended maximum values. Improving ventilation lowered the CO_2_ concentration, resulting in a lower risk of airborne infections. Conclusions: The ventilation in many schools is not adequate to guarantee good indoor air quality. Ventilation is an important measure for reducing the risk of airborne infections in schools. The most important effect is to reduce the time of residence of pathogens in the classrooms.

## 1. Introduction

In Germany, there are about 32,228 schools, around half of them primary schools. During the 2020/2021 school year, 790,608 teachers taught about 8.38 million students at general education schools [1]. Many individuals of different age groups spend several hours together every weekday in relatively small areas in educational facilities. In connection with the Severe Acute Respiratory Syndrome Corona Virus 2 (SARS-CoV-2)—the cause of COVID-19 that was declared a pandemic by the WHO on 11 March 2020 [2]—schools attracted attention as potential hotspots for the transmission of SARS-CoV-2. As a result, schools in Germany were closed in March 2020 as part of a nationwide lockdown to reduce the further spread of SARS-CoV-2 and the infection of families [3]. Certainly, these measures prevented many infections. However, there were various side effects, such as deterioration in school performance, psychological and physiological illness, and violence in homes, not to mention economic costs, which will need to be prevented in the future [4,5,6,7]. SARS-CoV-2 is transmitted primarily via infectious droplets and aerosols produced when speaking, breathing, coughing, and sneezing [8,9,10,11,12]. As far as is known, contact transmission, by means of contaminated surfaces or objects, plays only a minor role [13]. Aerosols spread in a room and can persist for longer periods, especially when air exchange is limited. They remain potentially infectious so that there is also a more widespread risk of infection in the far field (more than 1.5 m from an infectious person). In the case of droplet infection, on the other hand, transmission tends to take place between individuals in closer proximity, in the near field (in a radius of about 1.5 m from an infectious person). Airborne infections through droplets and aerosols can, however, merge so that a strict distinction is either difficult to make or is not useful [14].

There are other pathogens that are not as well known to the public but which can also lead to local outbreaks in a school setting. Important examples are respiratory pathogens such as the influenza virus [15,16], the measles virus [17], or the mycobacterium tuberculosis [18].

Improving ventilation can reduce the transmission of airborne pathogens by diluting or eliminating pathogens [11,19]. The ventilation can be natural ventilation (NV), for example through windows/doors, or mechanical ventilation (MV), for example by heating, ventilation, and air conditioning (HVAC) systems. A combination of NV and MV in the form of hybrid ventilation is also possible [20]. Most European schools are ventilated by natural ventilation without a defined ventilation rate [21,22].

Carbon dioxide (CO_2_) is exhaled together with droplets/particles that can contain virus. Indoor CO_2_ concentration is often used as an indicator of indoor air quality (IAQ) and the available ventilation rate per person [23], and is therefore often used as a surrogate parameter for the risk of infection or transmission of SARS-CoV-2 or other airborne infectious pathogens [24,25,26]. In Germany, indoor CO_2_ concentrations below 1000 ppm are classified as harmless, concentrations between 1000 and 2000 ppm as conspicuous, and concentrations over 2000 ppm as unacceptable [27]. It is possible that revised CO_2_ limit values are necessary, related to activity levels [26]. Originally, von Pettenkofer proposed the reference value of 1000 ppm as the upper limit for CO_2_ concentration indoors [28]. When proposing this reference value, he intended primarily to prevent students from having problems concentrating because of excessive concentrations of CO_2_. It is relatively easy and comparatively cheap to measure CO_2_ concentration using CO_2_ measurement equipment.

There are some limitations to using the CO_2_ concentration as a surrogate parameter for the risk of infection: after a certain amount of time, it reaches a steady state, whereas the number of particles containing virus that are inhaled by a person in the room increases over time even if the concentration of particles in the room remains unchanged. Kriegel et al. postulate that the CO_2_ dose (ppm*h) might be more meaningful than the CO_2_ concentration when estimating the risk of infection [29].

After more than 2.5 years of pandemic experience we want to examine, on the basis of published field studies, whether and to what extent interventions in relation to ventilation measures in schools have contributed to reducing the risk of airborne infection or to CO_2_ concentration, the surrogate endpoint. Additional measures such as masks, regular testing, vaccinations, etc., which can also reduce the risk of infection, were not investigated [16,30,31,32,33].

## 2. Methods

### 2.1. Search Strategy

Systematic searches of the literature in the databases Embase, MEDLINE, and ScienceDirect were carried out by two persons between 9 July 2021 and 6 May 2022. Publications in English or German, with a publication date previous to 1 May 2022 were considered. To identify relevant studies in the literature, a combination of the following keywords was used: “school”, “classroom”, “child”, “student”, “pupil”, “ventilation”, “CO_2_”, “air filtration”, “indoor air quality”, “architecture”, “building”, “COVID-19”, “SARS-CoV-2”, “measles”, “respiratory syncytial virus”, “infection”, “prevention”, and “airborne transmission”. In addition, we considered relevant publications found during the study of publications identified earlier. The program Endnote was used for reference management and the elimination of duplicates.

### 2.2. Inclusion and Exclusion Criteria

Studies were included that were carried out in classrooms or school buildings with the primary endpoint CO_2_ concentration or the risk of infection/transmission of various airborne pathogens (e.g., SARS-CoV-2, measles, influenza) or infection from these airborne pathogens in relation to ventilation or building-associated factors. “School” here, depending on the country where a study was carried out, refers to K-12 schools or pre-schools, or primary and secondary schools. Colleges and universities, frequently with larger classroom designs, are not considered. In addition, only studies carried out in high and middle-income countries in climate zones comparable to Germany’s were included in order to ensure comparability and transferability. Regarding the study design, intervention studies, observational studies, and mathematical modeling studies were included. In the course of the writing this article, a new study was published that was highly relevant to the question being investigated [34]. This study was also included, although its publication date was later than the period used in literature search.

There was a great deal of overlap among studies carried out before the pandemic which examined the effects of CO_2_ in classrooms, e.g., as a surrogate parameter for the occurrence of concentration disorders. Hence, observational and mathematical modeling studies published before 2020, in which CO_2_ concentration was not associated with the transmission of airborne infections as their primary endpoint, were excluded. 

### 2.3. Study Selection and Structuring

**Figure 1:**
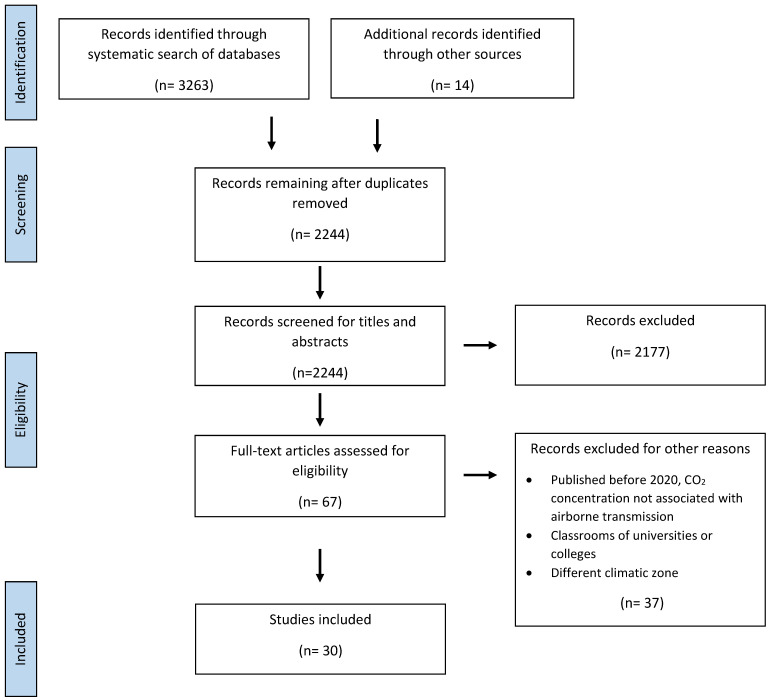
Flowchart of study selection process.

In selecting studies, after duplicates were eliminated, studies were screened by title and abstract. The remaining studies were then read in full and checked for relevance. A flowchart depicting the process of study selection is shown in Figure 1. 

## 3. Results

We identified 30 studies that met the inclusion criteria (Figure 1). Of these, six were intervention studies whose primary endpoint was CO_2_ concentration or SARS-CoV-2 infection in clusters of cases (Table 1), 16 were observational studies, some with additional mathematical modeling, and eight were mathematical modeling studies whose primary endpoints were CO_2_ concentration or infection by/transmission of various respiratory pathogens, e.g., SARS-CoV-2, measles, and influenza (Table 2). 

In summary, in many classrooms, CO_2_ concentrations were higher than 1000 ppm and ventilation could lower CO_2_ concentrations [24,34,35,36,37,38]. Nevertheless, even this step was sometimes not adequate to keep CO_2_ concentrations below 1000 ppm permanently, especially when individuals were present in the room during the ventilation period [39]. As shown in other studies [40,41,42,43], CO_2_ concentrations in classrooms with mechanical ventilation were lower than in those naturally ventilated. For example, Vassella et al. found the median CO_2_ concentration in MV classrooms was 686–1320 ppm, whereas in NV classrooms it was 862–2898 ppm [38]. In one intervention study, the authors found that in mechanically ventilated classrooms, the relative risk of infection with SARS-CoV-2 was reduced by at least 74% compared with those naturally ventilated. At higher ventilation rates of > 10 L s^−1^ student ^−1^, the relative risk of infection decreased by at least 80%. The protective effect of MV was greater in periods of higher regional incidence of SARS-CoV-2 [34]. 

Some building-associated factors can influence the efficiency of ventilation and the risk of infection by airborne pathogens. Room size affected the risk of infection, to a particular degree in small, poorly ventilated rooms [22,44]. Stein-Zamir et al. describe a major SARS-CoV-2 outbreak triggered by two index cases. In the school in question, classrooms were overcrowded (1.1–1.3 m^2^ per person). The requirement to wear masks had nonetheless been abolished and contacts between students also existed outside the school setting, possibly leading to infections outside the school [45]. 

A visual feedback system that monitored CO_2_ concentrations and indicated the need for ventilation could achieve a considerable reduction in CO_2_ concentrations through increased NV as compared to the control group without a visual feedback system [35].

**Table 1:** Intervention studies.

Reference	Study Type	Setting	Methods	Primary Endpoint	Main Results	Side Effects
[24]	Intervention study	11 classrooms, (9 pre-school, primary and secondary schools), Italy, Jan–Feb 2021	NV regime. Questionnaire to evaluate occupancy and general ventilation behavior. (1) Control: ventilation as usual. (2) Intervention: door always open, windows open for 10 min during break and when CO_2_ conc. reaches 700 ppm. Additional measures: use of hand sanitizer, cleaning of surfaces, wearing masks, keeping distance.	CO_2_ concen-tration	(1) Mean CO_2_ conc.: 721–1325 ppm. 54% of the classrooms had mean CO_2_ conc. > 1000 ppm. Maximum CO_2_ conc.: 867–3947 ppm. (2) 91% of classrooms had mean CO_2_ conc. < 1000 ppm, 36% had CO_2_ conc. < 700 ppm. Real time visualization of CO_2_ conc. better than merely following systematic ventilation protocols. In some classrooms, improved NV was not adequate to achieve good air quality because of structural building elements.	Low temperatures despite the use of radiators
[35]	Intervention study	4 classrooms, 1 elementary school, Denmark, 2 weeks in Mar-Apr 2011 and Jun 2011 each	Visual CO_2_ feedback, colors representing specific CO_2_ conc. indicating the need to ventilate (NV). Building with mixing-type MV system. In half of the classrooms the MV system was turned off during season when rooms are heated, measurements were performed one week with visual feedback alternating with one week without visual feedback in all classrooms (cross-over method). During season when rooms are cooled, measurements were performed for 2 weeks either with or without visual feedback in each half of the classrooms.	CO_2_ concen-tration	Before the intervention: CO_2_ maximum values up to 1500 ppm. During heating season: windows opened more often and CO_2_ conc. were lower in intervention group with visual feedback (below or around 1000 ppm vs. conc. up to around 1900 ppm in control group). In the cooling season: no difference in the frequency of opening windows with the visual feedback in classrooms and without mechanical cooling. In classrooms with mechanical cooling, windows were opened more often when visual feedback was used.	Estimated annual heating 15–23% higher, estimated annual cooling 18% lower in classrooms with visual CO_2_ feedback system.
[37]	Intervention study	81 classrooms 20 primary schools, Netherlands, Oct–Dec 2004 and Jan–Mar 2005	CO_2_ measurements taken before, immediately after, and 6 weeks after interventions: (1) Class-specific NV ventilation advice. (2) Class-specific advice and device warning (visual sign) when CO_2_ conc. > 1200 ppm. (3) Class-specific advice and teaching package. (4) Control group.	CO_2_ concen-tration	Before interventions: CO_2_ conc. > 1000 ppm in 64% of the school day. (1) No improvement of ventilation behavior significantly in the longer term. (2) In the short term fewest periods with CO_2_ conc. > 1000 ppm compared to other groups. (3) > (2) Long term improvement of ventilation situation, CO_2_ conc. > 1000 ppm in 40% of the school day.	
[38]	(1) Cross-sectional study (2) Intervention study	(1) 100 classrooms, 96 Swiss primary and low secondary schools(2) 19 (+4) classrooms, during season when rooms are heated	(1) Standard ventilation as usual, NV in 94% of classrooms. (2.1) Strategic NV during breaks and before/after lessons (rooms unoccupied). Written and oral instructions to teach ventilation behavior. Interactive simulation tool to develop ventilation plan used in 4 classes to develop specific ventilation strategy. (2.2) Control group: Same 19 classrooms as (1) with previous measurements.	CO_2_ concen-tration	Average percentage of lessons with CO_2_ conc. < 1000 ppm increased from 18% to 42% as a result of intervention. (1) More than 2/3 of classrooms had CO_2_ conc. > 2000 ppm. MV: Median CO_2_ conc.: 686–1320 ppm; maximum median: 1364 ppm. NV: Median CO_2_ conc.: 862–2898 ppm; maximum median: 2754 ppm. (2.1) Median CO_2_ conc.: 1097 ppm; median maximum conc. decreased to 1892 ppm. (2.2) Median CO_2_ conc.: 1600 ppm. Higher CO_2_ conc. with the number of consecutive lessons in (1) and (2).	
[46]	Intervention study	18 classrooms, 17 primary schools, Netherlands periods when rooms were heated, 2010–2012	(1) Intervention group (12 classrooms): week 1: standard ventilation; week 2/3: ventilation with mobile MV device; target CO_2_ conc.: 800 or 1200 ppm for 1 week at a time, cross-over design. Preheated outside air was introduced and air was recirculated. (2) Control group (6 class-rooms): NV, no specific ventilation strategy.	CO_2_ concen-tration	(1) Mean CO_2_ conc.: 1399 ppm (SD: 350) in week 1, decreased in week 2 and 3 to mean CO_2_ conc. of 841 ppm (SD: 65, target set 800 ppm) and mean CO_2_ conc. of 975 ppm (SD: 73, target set 1200 ppm). More stable CO_2_ conc. (2) Week 1: mean CO_2_ conc. 1208 ppm (SD: 244); week 2/3: mean CO_2_ conc. 1350 (SD: 486).	
[34]	Intervention study	10,441 classrooms, 1419 schools, Italy, September 2021–January 2022	316 classrooms in 56 schools with MV (single room ventilation units, most with filters and heat recovery), 205,247 students. Additional measures (masks, distancing, increased NV). MV turned on before start of school, operating throughout school day. Maximum air flow rates 100–1000 m^3^ h^−1^ corresponding to VRs per person of 1.4–14 L s^−1^ student ^−1^. (1) Intervention: Installation of MVS in classrooms (2) Classrooms with NV. Extrapolation of temporal exposure from regional weekly SARS-CoV-2 incidence; relative risk reduction correlated with presence of MVSs in classrooms.	SARS-CoV-2 infection of clusters of cases (≥2 cases until December 2021; ≥3 from January 2022)	(1) 31 infected students (2) 3090 infected students in clusters. Monthly incidence proportion (IP = number of cases/1000 students): increased from 13 September to 23 December 2021 and especially from 7–31 January 2022 (Omicron), lower values in MV classrooms (4.9. vs. 15.3 in NV). Incidence proportion ratio (IPR = ratio between IP in classrooms with and without MV): 0.32. Protective effect of MV greater with higher regional incidence. Greater relative risk reduction (RRR) with higher ventilation rate. In the most conservatively calculated scenario: in total 74% RRR with MV vs. NV; 80% RRR with VR > 10–14 L s^−1^ student ^−1^. For each additional unit of VR per person, the RRR ranged from 12–15%. This association was significant irrespective of occupancy, educational level, and location.	

Note: NV = natural ventilation; MV = mechanical ventilation/mechanically ventilated; MVS = mechanical ventilation system; SD = standard deviation; CO_2_ conc. = CO_2_ concentration; RR = relative risk; RRR = relative risk reduction; IP = incidence proportion; IPR = incidence proportion ratio.

**Table 2:** Observational studies and mathematical modeling studies.

Reference	Study Type	Setting	Methods	Primary Endpoint	Main Results	Side Effects
[36]	Observational study	(1) 9 secondary schools, Spain, December 2020–January 2021. (2) 3 classrooms, 1 secondary school, heating period before and during pandemic.	(1) Survey/interviews on (building) characteristics, heating consumption and thermal comfort. (2) CO_2_ measurements. (1) and (2) During pandemic: Cross ventilation after each class or at the beginning of the day, during 30 min break, at end of day, and sometimes during classes. Before pandemic: brief individual ventilation periods.	CO_2_ concen-tration	(2) Reduction of mean CO_2_ conc. from 2478 ppm (SD: 852) to 1105 ppm (SD 295). The increase of CO_2_ conc. during school hours decreased from 857 ppm per hour to 135 ppm per hour. CO_2_ conc. fluctuated less.	(1) and (2) Mean indoor temperature: 18 °C, decrease of 2 °C. Increased heating use 9–40%.
[44]	Observational study	3 classrooms, 1 primary school, Germany Apr–May 2022	NV for 5 min every 20 min during lessons vs. no ventilation. Reduced occupancy.	CO_2_ concen-tration	CO_2_ conc. < 1000 ppm can be achieved through natural cross ventilation. No ventilation: almost linear increase in CO_2_ conc.	
[47]	Observational study	50 classrooms, 2 K-12 schools, USA, Jan–Mar 2021	Measurement of CO_2_ conc. after controlled release in different scenarios.	CO_2_ concen-tration	Increase of ACH, especially with natural cross ventilation. ACH > 5/h in 90% of classrooms with ventilation vs. ACH < 3/h without ventilation.	
[48]	Observational study	19 classrooms, 7 pre-school, primary or secondary schools, Spain, Sept–Oct 2020	Measurement with natural cross ventilation continuously during classes and breaks. In some classes, masks were worn. 1 room equipped with additional MV.	CO_2_ concen-tration	26% of the classrooms had CO_2_ conc. > 700 ppm. Better ventilation in preschools: average CO_2_ conc. 553 ppm, SD 56, max. 1075 ppm. Primary schools: average CO_2_ conc. 602 ppm, SD 109, max. 1341 ppm. Secondary schools: average CO_2_ conc. 699 ppm, SD 172, max. 2117 ppm.	
[39]	Observational study	9 classes, 1 classroom, 1 secondary school in Latvia, September 2020	NV. CO_2_ measurements during teaching hours and breaks, additional questionnaire. No details about frequency or duration of ventilation. Students usually remained in classrooms during breaks.	CO_2_ concen-tration	Average CO_2_ conc. about 2380 ppm, maximum 4424 ppm. Higher CO_2_ conc. in 3rd and 4th periods, probably due to shorter breaks in the morning. During breaks, CO_2_ conc. decreased slightly and increased rapidly after breaks.	Average temperature 22 °C, min: 18.5 °C.
[49]	Observational study	2 classrooms, 1 elementary school, Spain, (1) Jan–Mar 2020 before pandemic (2) Nov 2020–Jan 2021	MV system, measurement of CO_2_ concentration. (1) Sometimes additional NV. (2) MV sometimes turned off, continuous NV following COVID-19 protocols.	CO_2_ concen-tration	(1) Mean CO_2_ conc. 1033 ppm (range 618–1571) or 1079 ppm (range 530–1726) in both classrooms. (2) CO_2_ conc. 604 ppm (range 466–781) or 740 ppm (range 514–1177).	(2) Lower indoor temperature, more frequent thermal discomfort
[50]	Observational study	2 classrooms, 1 school, Germany, heating period before and during pandemic	Measurements without ventilation and after opening of up to 5 windows and door (NV).	CO_2_ concen-tration	CO_2_ conc. ranging between 2500–2800 ppm after a school lesson with no specific ventilation. After several minutes of NV, CO_2_ conc. around 1000 ppm.	
[51]	Observational Study	2 K-12 schools, USA, fall 2020	Detection of SARS-CoV-2 cases in 2 schools after implementation of various mitigation strategies (e.g., MERV filters, increased ventilation, social distancing, routine testing, masks). No direct comparison of the effect of the different strategies.	SARS-CoV-2 infection	School A: 109 positive cases (4.9%), R_0_ 0.49; school B: 25 positive cases (2.0%), R_0_ 0.02. 9% of cases responsible for identified clusters. 72% of the cases transmitted in school were associated with noncompliance, many cases of transmission outside school setting.	
[52]	Observational study, outbreak analysis	1 secondary school, Germany, 2020	Analysis of an outbreak after schools reopened after the first lockdown. Examination of causes and course (clinical, contact, laboratory data, WGS analysis). Students did not wear masks, teachers sometimes wore masks.	SARS-CoV-2 infection	A teacher was identified as the index case, subsequently 31 students, 2 teachers and 3 household contacts were infected. Most infections were in connection with 2 lessons of the index case (1 building, rooms of possible transmission were all located on two floors). Limited ventilation, narrow sanitary facilities, 1 crowded classroom.	
[17]	Observational study, outbreak analysis	1 elementary school, upstate New York, USA, 1974	Analysis of a large measles outbreak, investigation of the impact of vaccination and ventilation. School equipped with 2 ventilation systems. Air is recirculated after filtration.	Measles Infection	97% of the children were vaccinated. Index case infected 28 other students, 60 children were subsequently infected. Recirculation of the virus by the ventilation system augmented transmission. The most important exposure sites were the same classroom as the infector(s), another classroom that used the same ventilation system, and school buses.	
[45]	Observational study/Case study	1 school, Israel, May 2020	Analysis of a SARS-CoV-2 outbreak in a school 10 days after reopening. Air conditioning systems in operation (separate for each classroom).	SARS-CoV-2 infection	153 students (13.2%) and 25 staff members (16.6%) tested positive after detection of 2 positive index cases. Due to heatwave no masks worn, crowded classes (1.1–1.3 m^2^ per person), extra-curricular activities. Also contacts on way to school.	
[53]	Observational study, mathematical modeling study	45 classrooms, 11 primary and secondary schools, England, Nov 2015–Mar 2020	Hybrid ventilation systems. No specific ventilation strategy. CO_2_ measurement and calculation of infection risk and secondary infections, for two periods (5 days) in (1) Jan and (2) July 2018.	CO_2_ concen-tration, SARS-CoV-2 infection risk	(1) Average CO_2_ conc. around 1500 ppm, short periods with max. conc. > 2000 ppm. (2) CO_2_ conc. half of those in (1) due to warmer temperatures and increased ventilation. Infection risk in (1) about twice that in (2). Variations of secondary infections between the classrooms, even those using the same ventilation system.	
[54]	(1) Observational study (2) mathematical modeling study	4 classrooms, 2 high schools, Italy, winter 2015/2016	(1) NV with different ventilation scenarios (2) Simulation: MV with different ACH, normal occupancy. Recommended CO_2_ conc.: max. 700 ppm higher than outdoor concentration.	CO_2_ concentration, SARS-CoV-2 infection risk	(1) Frequent, short ventilation periods efficiently reduce CO_2_ conc., but recommended maximum conc. were not guaranteed permanently. Rapid decrease/increase of CO_2_ conc. during/after ventilation. Maximum CO_2_ conc.: 5136 ppm (school 1). Continuous increase up to 4680 ppm without ventilation (school 2). Infection risk > 1% even when using additional filtering methods.(2) higher ACH reduced infection risk from 23% (8 L s^−1^ person^−1^) to 7.2% (32 L s^−1^ person^−1^) with additional filtration (efficiency 95%): 0.38%.	Decrease of indoor temperature, thermal discomfort. Energy consumption can be reduced up to 72% using a “High Energy Air Handling Unit“ with thermal recovery.
[30]	Observational study, mathematical modeling study	101 classrooms, 19 elementary schools, USA, Dec 2017–Sept 2018	CO_2_ conc. were measured during the heating and the cooling seasons. MV in 37% of the schools. 18% had either no windows or windows that could not be opened. Certain ventilation strategies were not applied.	CO_2_ concentration, SARS-CoV-2 transmission risk	No significant differences in CO_2_ conc. between cooling (mean 990, range 430–2200 ppm) and heating seasons (mean 980 ppm, range 510–1900 ppm). Transmission risk was higher during heating season (increase of 28%). It was lower in classrooms with MV (risk 0.059 vs. 0.081 in NV). Higher transmission risk from teacher to student (mean conc. 0.20/0.35) than from student to teacher (0.14/0.26) or from student to student (0.046/0.091) with mask/without mask.	
[55]	Observational study, mathematical modeling study	3 classrooms, 1 elementary school, South Korea, May 2020	Measurement of CO_2_ decay by cross vs. single-sided ventilation with 0%, 15%, 30% and 100% window opening ratio. Air conditioner in operation during ventilation (set at 25 °C). Measurements when unoccupied. Infection risk calculated for different scenarios with 0.5 h to 3 h exposure time.	CO_2_ concentration, SARS-CoV-2 infection risk	Cross ventilation resulted in higher average ventilation rates (6.38/h (15% opening ratio), 10.53/h (30% opening ratio), 22.39/h (100% opening ratio) than single-sided ventilation 2.13/h (15% opening ratio), 2.90/h (30% opening ratio). VR reduced when air conditioner in operation. Without ventilation, infection risk >1% even with mask and exposure time of 0.5 h. Infection risk <1% with cross ventilation without mask with 30% window opening and 1 h exposure. With single sided ventilation, infection risk of <1% can only be achieved with masks and exposure time of max. 1 h.	Possible risk of cross transmission with strong indoor airflow. 10.2% and 22.5% higher energy consumption (windows opening ratio 15% and 30% vs. 0%).
[22]	Mathematical modeling study Additional exemplary observation	1 classroom, 1 high school, Italy, June 2021	CO_2_ measurement and modeling of infection risk for different ventilation and room scenarios. NV primarily during breaks. Models with/without both masks and teacher’s use of a microphone.	CO_2_ concen-tration, SARS-CoV-2 infection risk	70–80% reduction of infection risk in log scale by NV. Reduction in intensity with which teachers speak using a microphone: additional 20% risk reduction (without masks) almost 40% (with masks). Increasing total area of the classroom cuts infection risk almost in half.	
[56]	Survey, mathematical modeling study	169 Elementary and K-5 schools, Georgia, USA Nov–Dec 2020	Survey of different prevention strategies: increased NV, air filtration, masks, physical distancing, barriers on school desks, cohort size. Association of SARS-CoV-2 cases with prevention strategies was calculated.	SARS-CoV-2 infection	35% lower incidence when schools improved their ventilation strategies, 48% reduction with combination of increased NV and air filtration/purification and 37% reduction when students and staff wore face masks.	
[57]	Mathematical modeling study	111,485 public and private schools, USA	Estimation of occupant density in 1433 representative schools. Simulation of infection risk for two scenarios: one year pandemic scenario and epidemiological scenario, each with different infection prevention strategies. Assumed baseline ventilation rate: 2 ACH.	SARS-CoV-2 infection risk	90% of schools with infection risk >1%; Dec: 6.83%, July: 3.85%. Infection risk can be lowered by 16.5% by increasing the VR from 2/h to 2.5/h and by 8% by increasing the VR from 5.5/h to 6/h. Reduction using (MERV13) filters and by reduction of occupancy. To achieve an infection risk <1%, a combination of intervention strategies is required. Effectiveness of prevention strategies depended on school characteristics and pandemic periods.	Increased energy costs when using better MERV filters or higher VRs.
[58]	Mathematical modeling study	Different indoor spaces, among others, K-12 schools	Modeling of SARS-CoV-2 infection risk in different locations with different indoor air quality (IAQ) strategies.	SARS-CoV-2 infection-/transmission risk	Higher probability (mean, SD) that teacher spreads virus (13.2%, 12.0) than student to student (3.8%, 3.6). Higher infection risk in dining areas (10.1%, 8.9) and gym (8.3%, 7.7) than in library (0.3%, 0.2) due to lower occupancy, relatively better ventilation. Reduction of infection risk: when doubling total supply airflow rate: 37% reduction, 100% outdoor air, or HEPA filter: 27% reduction, displacement ventilation: 26% reduction, partitions: 46% reduction, personal ventilation: 46% reduction.	High costs of implementation and maintaining certain IAQ strategies.
[59]	Mathematical modeling study	Various scenarios, including classrooms	Calculation of required VRs in order to obtain an infection risk of <1% for various scenarios. Typical classroom (348 m^3^) with exposure time of 2 h.	SARS-CoV-2 infection risk	Required VR per infector is 100–350 m^3^/h (0.25 h exposure time) and 1200–4000 m^3^/h (3 h exposure time) without masks and VR of 30–90 m^3^/h (0.25 h exposure time) and 300–1000 m^3^/h (3 h exposure time) with masks. For a typical classroom, ACH of 4.8–15/h or 1.2–3.5/h are necessary to obtain an infection risk <1% (without or with masks respectively). These VR can be achieved using a normal MV system or NV for all scenarios.	
[60]	Mathematical modeling study	Various spaces in public buildings, incl. school classrooms	Calculation of the infection probability for specific rooms and calculation of VR required to achieve a specific probability of infection (with and without masks).	SARS-CoV-2 infection risk	Lower infection probability is easier to achieve in larger rooms, but usually there are more susceptible persons present. Example classrooms: 32 m^2^, AER 3.68/h, infection probability 0.034. 48 m^2^, AER: 4.48/h, infection probability 0.019. The total flow rate per infected person is essential in order to reduce the probability of infection.	Increased energy consumption (for MV).
[61]	Mathematical modeling study	Typical classroom, 1 high-school	Simulation of different scenarios (e.g., different infectors, intensity of speaking) with 1 infector and only airborne virus transmission using MV or NV. Calculation of required AER and ventilation procedures to obtain a transmission <1 during lessons, corresponding to an individual 4.2% risk of infection. 5 h school time.	CO_2_ concen-tration, SARS-CoV-2 and seasonal influenza infection risk	CO_2_ conc. reaches an equilibrium of 750 ppm after 30 min (MV, AER 9.5/h). A maximum CO_2_ concentration as indicator of transmission can be misrepresentative (due to dynamics). Required AER needed to prevent a seasonal influenza infection: <0.1/h, achieved for all scenarios; to prevent a SARS-CoV-2 infection: 9.5/h and 0.8/h (teacher infector, 60 min loud speaking vs. muted speaking through microphone). Required AER (student as infector) dependent on speaking/breathing time and attendance in classes: 0.8–3.5/h. Long ventilation periods or high AER sometimes not realizable with NV. With NV useful to apply a feedback control strategy with continuous CO_2_ measurements and adjusted ventilation times.	
[16]	Mathematical modeling study based on measurements in real classes	21 classrooms, 1 elementary school (including 2 kindergarten classrooms), Taiwan	Mechanical fans in elementary school classrooms, air conditioning system in kindergarten classrooms. No mention of additional NV. Class duration 40 min with 5–10 min breaks.	Pandemic influenza transmission risk, infection risk	Elementary school children have an infection probability of 0.56–0.64 and R_0_ values between 16.11–16.09 (age-dependent). Staff (25–45 years of age) have an infection risk of 0.07 and R_0_ of 2.80. The transmission potential can be reduced by implementing a higher ACH: R_0_ = 11.38/7.10/5.10/9.97 for 0.5/1/1.5/2/h ACH for kindergarten children. Vaccination as the most effective measure, combination of measures further reduce transmission risk.	
[62]	Mathematical modeling study	Primary and secondary schools, USA	Combination of a multi-zone Wells-Riley model, nationwide representative school building archetype model (with basic infection control scenario, regular and advanced ventilation-related control scenario) and a Monte-Carlo Simulation for estimating transmission risk. Estimates were validated with real outbreak data.	Measles transmission risk	Transmission risk 74 times higher for unvaccinated students, higher in high schools than in elementary schools (median 5.8% and 3.8% respectively). Schools with ductless systems without air filters have the highest transmission risk (median 6.0%), schools with ductless systems with air filters have the lowest (median: 3.7%). Using a better filter reduced transmission risk for unvaccinated students (45% for MERV8, 32% for MERV13, and 29% for HEPA filter, median values). Increasing ventilation rates decreased transmission risk for unvaccinated students (46% basic control scenario, 38% regular, 33% advanced infection control scenario).	

Note: NV = natural ventilation; MV = mechanical ventilation/mechanically ventilated; SD = standard deviation; ACH = air changes per hour; CO_2_ conc. = CO_2_ concentration, VR = ventilation rate.

## 4. Discussion

Improving ventilation in classrooms by means of mechanical or natural ventilation decreased CO_2_ concentrations and thus the assumed risk of infection [24,34,35,37,38]. In some studies, however, CO_2_ concentrations were still above the recommended upper limit of 1000 ppm [37]. Despite the large number of studies found in the literature search, only six intervention studies were identified that met the inclusion criteria. In addition, the studies were very heterogeneous with regard to the building architecture (size of the classrooms, number, size, arrangement and orientation of the windows, etc.) and setting (country, season). In some studies, several infection prevention measures were applied simultaneously, which complicated a determination of the extent of the effect of a specific measure. 

The literature search did not enable us to define maximum acceptable values for CO_2_ concentration. However, because it is often recommended by other authors and organizations [63,64,65], we postulate that the indoor CO_2_ concentration should not exceed 1000 ppm on average over time in all classrooms. During a pandemic involving an airborne pathogen, it should not exceed 800 ppm on average over time, although CO_2_ concentrations up to 1000 ppm for short periods are tolerable. This can be implemented using mechanical ventilation, for example, using HVAC systems, or by NV with windows and doors [66]. Our literature search confirms earlier findings that mechanically ventilated classrooms have significantly higher ventilation rates than naturally ventilated ones [40,41,42,43]. Thus, we also conclude that classrooms should be equipped with an MV system, since mechanically ventilated classrooms appear to have lower, more stable mean CO_2_ concentrations than naturally ventilated ones. Hence, a reduction of aerosols that could contain virus can be more easily achieved, which would result in a reduction of the risk of infection [30,34,38,46]. In one study, it was shown that a 74% reduction of the relative risk of infection could be achieved in classrooms with MV systems, and that for each additional unit in the ventilation rate per student, the relative risk reduction ranged from 12–15% [34]. The ventilation rates need to be adjusted depending on the age, activity, and number of individuals in the room. There are other positive effects of HVAC systems, such as indoor temperature regulation, which may prevent school closures due to extremely high temperatures. Birmili et al. found that, especially with extremely low or high outdoor temperatures, HVAC systems can prevent thermal discomfort [14]. Moreover, the elimination of CO_2_ and other possible pollutants may improve students’ ability to concentrate [67,68]. Installation or retrofitting of HVAC systems should be standard in schools. Until this is implemented, rooms should have a sufficient number of windows to enable large-area natural ventilation by means of a standardized ventilation regime. 

Miranda et al. found that a strong NV regime could keep the average CO_2_ concentrations at low levels between 450 and 650 ppm in university classrooms. However, the significant drop in indoor temperature led to thermal dissatisfaction [69]. Rooms that cannot be ventilated either naturally or mechanically are not suitable for school lessons. Like other authors [70,71] and organizations [72], we recommend installing CO_2_ measuring devices with clearly visible displays or sound alarms in classrooms. Such equipment indicates when ventilation is needed and helps to check the success of the ventilation. Several studies showed that CO_2_ concentration could be decreased dramatically using NV if CO_2_ measuring devices that visualized the effect of ventilation monitored CO_2_ concentration [24,35,37]. Laurent and Frans found that the use of CO_2_ measuring devices in a hospital resulted in significantly shorter periods of time with CO_2_ concentrations above 1000 ppm and lower overall maximum values [73]. A visual feedback system makes it easy to recognize when ventilation is necessary [35]. The REHVA recommends using CO_2_ monitors with red, yellow, and green indicator lights similar to a traffic signal [72].

As is already known from other studies, various environmental and building-related factors, (for example the difference between outside and inside temperature, the wind speed and direction, the arrangement/orientation of the windows, etc.) influence the efficiency of NV [22,50,74]. Due to the heterogeneity of the studies identified in our literature search, it was not possible to derive general recommendations about such environmental and building-related factors. Korsavi et al. suggest designers be aware of all contextual, occupant, and building-related factors and consider, for example, that an opening can have different airflow rates depending on the season and the outdoor conditions [75]. With NV, cross ventilation should be used, which is more effective than single-sided ventilation [55]. This is also recommended by Ferrari et al. [71] and was shown by Aguilar et al. to be true of university classrooms [76]. 

The use of (portable) air purifiers (APs) was controversial. The literature search turned up three studies which examined the influence of APs on aerosol concentration [44,50,77]. The endpoint “aerosol concentration” did not meet the inclusion criteria, thus these studies were not listed in Table 2 unless the CO_2_ concentration was also examined. In the three studies mentioned above, it was shown that it was possible to reduce the concentration of aerosol that could contain virus particles by means of air APs. It should be noted that these APs were equipped with HEPA filters. Air purification efficiency depends on, amongst others, the air purifier and filter class used. If it is not possible to guarantee the necessary air flow rates by means of MV or NV alone, an AP might be an ancillary measure for reducing the risk of infection. However, it should be kept in mind that APs only filter and recirculate air. Moreover, it is difficult to measure the effect of the air filtration during school hours. Although it is possible to conduct particle measurements, there are confounding factors, such as other sources of particles which are not emitted exclusively by human respiration which can influence the measurements. To guarantee good indoor air quality, the removal of “used air” and a supplementary supply of fresh outside air is still necessary to eliminate other (harmful) substances such as CO_2_ and other gaseous contaminants like volatile organic compounds.

Improving the ventilation situation can also have side effects. Frequent and long NV periods can cause a significant drop in interior temperatures, particularly in winter months, and thus result in thermal discomfort for those present [24,36]. Other side effects of NV may include noise and air pollution from neighboring streets or construction sites [48].

In addition to eliminating potentially infectious aerosols, the viral emission of individuals should be kept as low as possible. This depends on, among other things, the age, the intensity with which an individual speaks, and the type of activity of the individuals. In general, especially with the wild type of SARS-CoV-2, adults have higher viral emission than children do and in the context of schools, mainly the teacher speaks a lot and loudly [12,78,79,80,81,82,83]. The risk of transmission from teacher to student was greater than from student to teacher or from student to student in the studies identified [30,58]. The location of an infectious individual in the classroom can also influence the risk of infection. For example, the risk of inhaling a higher concentration of a pathogen was higher in close vicinity to the source individual, while the risk of infection decreased with increased distance from the infectious person [84,85,86]. For these reasons, we recommend that the distance between the teacher and the first row of school benches, in particular, should be large enough (at least 1.5 m) to reduce as much as possible the risk of infection to those in the near field of the teacher. Distance between students may further reduce the risk of infection, but due to limited classroom sizes, a large distance will not be possible in most classrooms. Since the teacher speaks the most and the loudest, the use of a microphone by the teacher can reduce the intensity of speech [22,61]. Other outbreak analyses have shown that transmission from teacher to teacher or from teacher to student had a large impact on outbreaks [52,87,88]. Likewise, Fleischer et al. postulate that particle emission by children is lower than by adults, possibly resulting in a lower risk of transmission by children. However individual/interpersonal variability of emission rates should be taken into consideration [79]. 

Improving ventilation can also reduce the transmission of other airborne pathogens. Du et al. (2020), for example, studied the impact of increased ventilation on tuberculosis outbreaks in poorly ventilated universities. As a result, maximum CO_2_ concentrations were reduced from approximately 3200 ppm to concentrations of approximately 600 ppm, and the incidence of tuberculosis in contact individuals was reduced by 97%. In summary, guaranteeing that CO_2_ concentrations do not exceed 1000 ppm could effectively control a tuberculosis outbreak in a university building [89]. 

## 5. Limitations

This review has several limitations. The number of intervention studies identified was small. Only studies published on or before 30 April 2022 were considered. Further studies have been published in the meantime which were not part of the review, with the exception of one that was highly relevant to this review. Similarly, other studies might have been found by expanding the keywords used in the literature search. In addition, we excluded observational and modeling studies published before 2020 whose primary endpoint was CO_2_ concentration unrelated to the transmission of airborne infections. The same applies to the exclusion of studies conducted in low-income countries in climate zones unlike Germany’s. Studies carried out in university classrooms were excluded due to room sizes, which are usually larger than school classrooms. Some results of such studies, however, might be applicable to school classrooms. CO_2_ concentration was chosen as a primary endpoint because it is often used as a surrogate parameter for estimating the risk of infection. It needs to be evaluated whether other parameters might be more appropriate (e.g., CO_2_ dose, relative humidity, temperature, etc.). Some of the study results were based on mathematical models whose estimates (e.g., the existence of a steady state or an even particle distribution in rooms) as well as specific values (e.g., quanta emission rate) were based on available data. The application of such models based on data of the wild type or early variants of SARS-CoV-2 to the current situation might be limited, as a result of the emergence of new SARS-CoV-2 variants and subsequent other individual emission rates and susceptibility. Our focus was on ventilation strategies as part of infection prevention measures. Hence, other measures, such as masks, regular screening tests, etc. were not discussed. 

## 6. Conclusions

The ventilation situation in many schools is not adequate to guarantee good indoor air quality. Ventilation is an important measure for reducing the risk of airborne infection in schools. It is most important to reduce the time of residence of pathogens in the classrooms. Schools should have a well-functioning mechanical or natural ventilation system in order to avoid airborne infections in general. Compliance with ventilation measures must be ensured, in particular during a pandemic, and ventilation measures may need to be intensified to further reduce risk of infection during school operations. 

## Data Availability

Not applicable.

## References

[1] (2022). Statistisches_Bundesamt. https://www.destatis.de/DE/Themen/Gesellschaft-Umwelt/Bildung-Forschung-Kultur/Schulen/_inhalt.html.

[2] WHO WHO Director-General’s Opening Remarks at the Mission Briefing on COVID-19—11 March 2020. https://www.who.int/director-general/speeches/detail/who-director-general-s-opening-remarks-at-the-media-briefing-on-covid-19--11-march-2020.

[3] Otte Im Kampe E., Lehfeld A.S., Buda S., Buchholz U., Haas W. (2020). Surveillance of COVID-19 school outbreaks, Germany, March to August 2020. Euro. Surveill..

[4] Kuhfeld M., Soland J., Tarasawa B., Johnson A., Ruzek E., Liu J. (2020). Projecting the Potential Impact of COVID-19 School Closures on Academic Achievement. Educ. Res..

[5] Nicola M., Alsafi Z., Sohrabi C., Kerwan A., Al-Jabir A., Iosifidis C., Agha M., Agha R. (2020). The socio-economic implications of the coronavirus pandemic (COVID-19): A review. Int. J. Surg..

[6] UNESCO Adverse Consequences of School Closures. https://en.unesco.org/covid19/educationresponse/consequences.

[7] ECDC (2021). COVID-19 in Children and the Role of School Settings in Transmission—Second Update. https://www.ecdc.europa.eu/en/publications-data/children-and-school-settings-covid-19-transmission.

[8] van Doremalen N., Bushmaker T., Morris D.H., Holbrook M.G., Gamble A., Williamson B.N., Tamin A., Harcourt J.L., Thornburg N.J., Gerber S.I. (2020). Aerosol and Surface Stability of SARS-CoV-2 as Compared with SARS-CoV-1. N. Engl. J. Med..

[9] Wang C.C., Prather K.A., Sznitman J., Jimenez J.L., Lakdawala S.S., Tufekci Z., Marr L.C. (2021). Airborne transmission of respiratory viruses. Science.

[10] Guo Z.-D., Wang Z.-Y., Zhang S.-F., Li X., Li L., Li C., Cui Y., Fu R.-B., Dong Y.-Z., Chi X.-Y. (2020). Aerosol and Surface Distribution of Severe Acute Respiratory Syndrome Coronavirus 2 in Hospital Wards, Wuhan, China, 2020. Emerg. Infect. Dis..

[11] Morawska L., Cao J. (2020). Airborne transmission of SARS-CoV-2: The world should face the reality. Environ. Int..

[12] Hartmann A., Lange J., Rotheudt H., Kriegel M. (2020). Emissionsrate und Partikelgröße von Bioaerosolen beim Atmen, Sprechen und Husten.

[13] ECDC (2022). Factsheet for health Professionals on Coronaviruses European Centre for Disease Prevention and Control. https://www.ecdc.europa.eu/en/factsheet-health-professionals-coronaviruses.

[14] Birmili W., Selinka H.C., Moriske H.J., Daniels A., Straff W. (2021). Ventilation Concepts in Schools for the Prevention of Transmission of Highly Infectious Viruses (SARS-CoV-2) by Aerosols in Indoor air. Bundesgesundheitsblatt Gesundh. Gesundh..

[15] Coleman K.K., Sigler W.V. (2020). Airborne Influenza A Virus Exposure in an Elementary School. Sci. Rep..

[16] Chen S.C., Liao C.M. (2008). Modelling control measures to reduce the impact of pandemic influenza among schoolchildren. Epidemiol. Infect..

[17] Riley R.L., Riley E.C., Murphy G. (1978). Airborne Spread of Measles in a Suburban Elementary-School. Am. Rev. Respir. Dis..

[18] Fang Y., Ma Y., Lu Q., Sun J., Pei Y. (2021). An outbreak of pulmonary tuberculosis and a follow-up investigation of latent tuberculosis in a high school in an eastern city in China, 2016–2019. PLoS ONE.

[19] Somsen G.A., van Rijn C., Kooij S., Bem R.A., Bonn D. (2020). Small droplet aerosols in poorly ventilated spaces and SARS-CoV-2 transmission. Lancet Respir. Med..

[20] Megahed N.A., Ghoneim E.M. (2021). Indoor Air Quality: Rethinking rules of building design strategies in post-pandemic architecture. Environ. Res..

[21] Baloch R.M., Maesano C.N., Christoffersen J., Banerjee S., Gabriel M., Csobod É., de Oliveira Fernandes E., Annesi-Maesano I. (2020). Indoor Air Pollution, Physical and Comfort Parameters Related to Schoolchildren’s Health: Data from the European SINPHONIE Study. Sci. Total Environ..

[22] Zivelonghi A., Lai M. (2021). Mitigating aerosol infection risk in school buildings: The role of natural ventilation, volume, occupancy and CO2 monitoring. Build. Environ..

[23] Shendell D.G., Prill R., Fisk W.J., Apte M.G., Blake D., Faulkner D. (2004). Associations between classroom CO2 concentrations and student attendance in Washington and Idaho. Indoor Air.

[24] Di Gilio A., Palmisani J., Pulimeno M., Cerino F., Cacace M., Miani A., de Gennaro G. (2021). CO2 concentration monitoring inside educational buildings as a strategic tool to reduce the risk of Sars-CoV-2 airborne transmission. Environ. Res..

[25] Rudnick S.N., Milton D.K. (2003). Risk of indoor airborne infection transmission estimated from carbon dioxide concentration. Indoor Air.

[26] Peng Z., Jimenez J.L. (2021). Exhaled CO2 as COVID-19 infection risk proxy for different indoor environments and activities. medRxiv.

[27] (2018). Ad-hoc-Arbeitsgruppe_Innenraumrichtwerte, Gesundheitliche Bewertung von Kohlendioxid in der Innenraumluft- Mitteilungen der Ad-hoc-Arbeitsgruppe Innenraumrichtwerte der Innenraumlufthygiene-Kommission des Umweltbundesamtes und der Obersten Landesgesundheitsbehörden. Bundesgesundheitsblatt Gesundh. Gesundh..

[28] Pettenkofer M.V. (1858). Über den Luftwechsel in Wohngebäuden.

[29] Kriegel M., Hartmann A., Buchholz U., Seifried J., Baumgarte S., Gastmeier P. (2021). SARS-CoV-2 Aerosol Transmission Indoors: A Closer Look at Viral Load, Infectivity, the Effectiveness of Preventive Measures and a Simple Approach for Practical Recommendations. Int. J. Environ. Res. Public Health.

[30] Pavilonis B., Ierardi A.M., Levine L., Mirer F., Kelvin E.A. (2021). Estimating aerosol transmission risk of SARS-CoV-2 in New York City public schools during reopening. Environ. Res..

[31] Donovan C.V., Rose C., Lewis K.N., Vang K., Stanley N., Motley M., Brown C.C., Gray F.J., Thompson J.W., Amick B.C. (2022). SARS-CoV-2 Incidence in K-12 School Districts with Mask-Required Versus Mask-Optional Policies—Arkansas, August-October 2021. MMWR Morb. Mortal Wkly. Rep..

[32] Falk A., Benda A., Falk P., Steffen S., Wallace Z., Hoeg T.B. (2021). COVID-19 Cases and Transmission in 17 K-12 Schools—Wood County, Wisconsin, August 31-November 29, 2020. MMWR Morb. Mortal Wkly Rep..

[33] Robert-Koch-Institut (2021). Die Impfung gegen COVID-19 in Deutschland zeigt eine hohe Wirksamkeit gegen SARS-CoV-2-Infektionen, Krankheitslast und Sterbefälle (Analyse der Impfeffekte im Zeitraum Januar bis Juli 2021). https://www.rki.de/DE/Content/Infekt/EpidBull/Archiv/2021/35/Art_01.html.

[34] Buonanno G., Ricolfi L., Morawska L., Stabile L. (2022). Increasing ventilation reduces SARS-CoV-2 airborne transmission in schools: A retrospective cohort study in Italy’s Marche region. Front Public Health.

[35] Wargocki P., Da Silva N.A. (2015). Use of visual CO2 feedback as a retrofit solution for improving classroom air quality. Indoor Air.

[36] Monge-Barrio A., Bes-Rastrollo M., Dorregaray-Oyaregui S., González-Martínez P., Martin-Calvo N., López-Hernández D., Arriazu-Ramos A., Sánchez-Ostiz A. (2022). Encouraging natural ventilation to improve indoor environmental conditions at schools. Case studies in the north of Spain before and during COVID. Energy Build..

[37] Geelen L.M.J., Huijbregts M.A.J., Ragas A.M.J., Bretveld R.W., Jans H.W.A., van Doorn W.J., Evertz S.J.C.J., van der Zijden A. (2008). Comparing the effectiveness of interventions to improve ventilation behavior in primary schools. Indoor Air.

[38] Vassella C.C., Koch J., Henzi A., Jordan A., Waeber R., Iannaccone R., Charriere R. (2021). From spontaneous to strategic natural window ventilation: Improving indoor air quality in Swiss schools. Int. J. Hyg. Environ. Health.

[39] Zemitis J., Bogdanovics R., Bogdanovica S. (2021). The Study of CO_2_ Concentration in A Classroom During the COVID-19 Safety Measures. E3S Web. Conf..

[40] Rodríguez D., Urbieta I.R., Velasco Á., Campano-Laborda M., Jiménez E. (2022). Assessment of indoor air quality and risk of COVID-19 infection in Spanish secondary school and university classrooms. Build. Environ..

[41] Scheff P.A., Paulius V.K., Huang S.W., Conroy L.M. (2000). Indoor air quality in a middle school, Part I: Use of CO_2_ as a tracer for effective ventilation. Appl. Occup. Environ. Hyg..

[42] Canha N., Mandin C., Ramalho O., Wyart G., Riberon J., Dassonville C., Hanninen O., Almeida S.M., Derbez M. (2016). Assessment of ventilation and indoor air pollutants in nursery and elementary schools in France. Indoor Air.

[43] Canha N., Almeida S.M., Freitas M.C., Täubel M., Hänninen O. (2013). Winter ventilation rates at primary schools: Comparison between Portugal and Finland. J. Toxicol. Environ. Health. A.

[44] Duill F.F., Schulz F., Jain A., Krieger L., van Wachem B., Beyrau F. (2021). The Impact of Large Mobile Air Purifiers on Aerosol Concentration in Classrooms and the Reduction of Airborne Transmission of SARS-CoV-2. Int. J. Environ. Res. Public Health.

[45] Stein-Zamir C., Abramson N., Shoob H., Libal E., Bitan M., Cardash T., Cayam R., Miskin I. (2020). A large COVID-19 outbreak in a high school 10 days after schools’ reopening, Israel, May 2020. Eurosurveillance.

[46] Rosbach J.T., Vonk M., Duijm F., van Ginkel J.T., Gehring U., Brunekreef B. (2013). A ventilation intervention study in classrooms to improve indoor air quality: The FRESH study. Environ. Health.

[47] McNeill V.F., Corsi R., Huffman J.A., King C., Klein R., Lamore M., Maeng D.Y., Miller S.L., Ng N.L., Olsiewski P. (2022). Room-level ventilation in schools and universities. Atmos. Environ. X.

[48] Villanueva F., Notario A., Cabañas B., Martín P., Salgado S., Gabriel M.F. (2021). Assessment of CO(2) and aerosol (PM(2.5), PM(10), UFP) concentrations during the reopening of schools in the COVID-19 pandemic: The case of a metropolitan area in Central-Southern Spain. Environ. Res..

[49] Alonso A., Llanos J., Escandón R., Sendra J.J. (2021). Effects of the COVID-19 Pandemic on Indoor Air Quality and Thermal Comfort of Primary Schools in Winter in a Mediterranean Climate. Sustainability.

[50] Curtius J., Granzin M., Schrod J. (2021). Testing mobile air purifiers in a school classroom: Reducing the airborne transmission risk for SARS-CoV-2. Aerosol. Sci. Technol..

[51] Gillespie D.L., Meyers L.A., Lachmann M., Redd S.C., Zenilman J.M. (2021). The Experience of 2 Independent Schools with In-Person Learning During the COVID-19 Pandemic. J. Sch. Health.

[52] Baumgarte S., Hartkopf F., Hölzer M., von Kleist M., Neitz S., Kriegel M., Bollongino K. (2022). Investigation of a Limited but Explosive COVID-19 Outbreak in a German Secondary School. Viruses.

[53] Vouriot C.V.M., Burridge H.C., Noakes C.J., Linden P.F. (2021). Seasonal variation in airborne infection risk in schools due to changes in ventilation inferred from monitored carbon dioxide. Indoor Air.

[54] Schibuola L., Chiara T. (2021). High energy efficiency ventilation to limit COVID-19 contagion in school environments. Energy Build..

[55] Park S., Choi Y., Song D., Kim E.K. (2021). Natural ventilation strategy and related issues to prevent coronavirus disease 2019 (COVID-19) airborne transmission in a school building. Sci. Total Environ..

[56] Gettings J., Czarnik M., Morris E., Haller E., Thompson-Paul A.M., Rasberry C., Lanzieri T.M., Smith-Grant J., Aholou T.M., Thomas E. (2021). Mask Use and Ventilation Improvements to Reduce COVID-19 Incidence in Elementary Schools—Georgia, November 16-December 11, 2020. MMWR Morb. Mortal Wkly. Rep..

[57] Xu Y., Cai J., Li S., He Q., Zhu S. (2021). Airborne infection risks of SARS-CoV-2 in U.S. schools and impacts of different intervention strategie. Sustain. Cities Soc..

[58] Shen J., Kong M., Dong B., Birnkrant M.J., Zhang J. (2021). A systematic approach to estimating the effectiveness of multi-scale IAQ strategies for reducing the risk of airborne infection of SARS-CoV-2. Build. Environ..

[59] Dai H., Zhao B. (2020). Association of the infection probability of COVID-19 with ventilation rates in confined spaces. Build. Simul..

[60] Kurnitski J., Kiil M., Wargocki P., Boerstra A., Seppänen O., Olesen B., Morawska L. (2021). Respiratory infection risk-based ventilation design method. Build. Environ..

[61] Stabile L., Pacitto A., Mikszewski A., Morawska L., Buonanno G. (2021). Ventilation procedures to minimize the airborne transmission of viruses in classrooms. Build. Environ..

[62] Azimi P., Keshavarz Z., Cedeno Laurent J.G., Allen J.G. (2020). Estimating the nationwide transmission risk of measles in US schools and impacts of vaccination and supplemental infection control strategies. BMC Infect. Dis..

[63] Clements-Croome D.J., Awbi H.B., Bakó-Biró Z., Kochhar N., Williams M. (2008). Ventilation rates in schools. Build. Environ..

[64] REHVA (2021). CO₂ Monitoring and Indoor Air Quality. https://www.rehva.eu/rehva-journal/chapter/co2-monitoring-and-indoor-air-quality.

[65] The_Lancet_COVID-19_Commission (2022). Proposed Non-infectious Air Delivery Rates (NADR) for Reducing Exposure to Airborne Respiratory Infectious Diseases. https://covid19commission.org/safe-work-travel.

[66] Umweltbundesamt (2017). Anforderungen an Lüftungskonzeptionen in Gebäuden Teil I: Bildungseinrichtungen. https://www.umweltbundesamt.de/sites/default/files/medien/1410/publikationen/uba_empfehlungspapier_lueftung_unterrichtsgebaeude_final_bf.pdf.

[67] Bakó-Biró Z., Clements-Croome D.J., Kochhar N., Awbi H.B., Williams M.J. (2012). Ventilation rates in schools and pupils’ performance. Build. Environ..

[68] Haverinen-Shaughnessy U., Moschandreas D.J., Shaughnessy R.J. (2011). Association between substandard classroom ventilation rates and students’ academic achievement. Indoor Air.

[69] Miranda M.T., Romero P., Valero-Amaro V., Arranz J.I., Montero I. (2022). Ventilation conditions and their influence on thermal comfort in examination classrooms in times of COVID-19. A case study in a Spanish area with Mediterranean climate. Int. J. Hyg. Env. Health.

[70] Kienbaum T. (2020). Hygienemanagement in Gesundheitseinrichtungen Teil 4: Effektives Lüften während der Pandmie. Hygienemanagement.

[71] Ferrari S., Blázquez T., Cardelli R., Puglisi G., Suárez R., Mazzarella L. (2022). Ventilation strategies to reduce airborne transmission of viruses in classrooms: A systematic review of scientific literature. Build. Environ..

[72] REHVA (2021). REHVA COVID19 Guidance version 4.1 How to operate HVAC and Other Building Service Systems to Prevent the Spread of the Coronavirus (SARS-CoV-2) Disease (COVID-19) in Workplaces. https://www.rehva.eu/fileadmin/user_upload/REHVA_COVID-19_guidance_document_V4.1_15042021.pdf.

[73] Laurent M.R., Frans J. (2022). Monitors to improve indoor air carbon dioxide concentrations in the hospital: A randomized crossover trial. Sci. Total Environ..

[74] de la Hoz-Torres M.L., Aguilar A.J., Ruiz D.P., Martínez-Aires M.D. (2021). Analysis of Impact of Natural Ventilation Strategies in Ventilation Rates and Indoor Environmental Acoustics Using Sensor Measurement Data in Educational Buildings. Sensors.

[75] Korsavi S.S., Montazami A., Mumovic D. (2020). Ventilation rates in naturally ventilated primary schools in the UK.; Contextual, Occupant and Building-related (COB) factors. Build. Environ..

[76] Aguilar A.J., de la Hoz-Torres M.L., Costa N., Arezes P., Martínez-Aires M.D., Ruiz D.P. (2022). Assessment of ventilation rates inside educational buildings in Southwestern Europe: Analysis of implemented strategic measures. J. Build. Eng..

[77] Burgmann S., Janoske U. (2021). Transmission and reduction of aerosols in classrooms using air purifier systems. Phys. Fluids.

[78] Asadi S., Wexler A.S., Cappa C.D., Barreda S., Bouvier N.M., Ristenpart W.D. (2019). Aerosol emission and superemission during human speech increase with voice loudness. Sci. Rep..

[79] Fleischer M., Schumann L., Hartmann A., Walker R.S., Ifrim L., von Zadow D., Luske J., Seybold J., Kriegel M., Murbe D. (2022). Pre-adolescent children exhibit lower aerosol particle volume emissions than adults for breathing, speaking, singing and shouting. J. R. Soc. Interface.

[80] Murbe D., Kriegel M., Lange J., Schumann L., Hartmann A., Fleischer M. (2021). Aerosol emission of adolescents voices during speaking, singing and shouting. PLoS ONE.

[81] Euser S., Aronson S., Manders I., van Lelyveld S., Herpers B., Sinnige J., Kalpoe J., van Gemeren C., Snijders D., Jansen R. (2022). SARS-CoV-2 viral-load distribution reveals that viral loads increase with age: A retrospective cross-sectional cohort study. Int. J. Epidemiol..

[82] Thompson H.A., Mousa A., Dighe A., Fu H., Arnedo-Pena A., Barrett P., Bellido-Blasco J., Bi Q., Caputi A., Chaw L. (2021). Severe Acute Respiratory Syndrome Coronavirus 2 (SARS-CoV-2) Setting-specific Transmission Rates: A Systematic Review and Meta-analysis. Clin. Infect. Dis..

[83] Viner R.M., Mytton O.T., Bonell C., Melendez-Torres G.J., Ward J., Hudson L., Waddington C., Thomas J., Russell S., van der Klis F. (2021). Susceptibility to SARS-CoV-2 Infection Among Children and Adolescents Compared with Adults: A Systematic Review and Meta-analysis. JAMA Pediatr..

[84] Zafarnejad R.-G., Paul M. (2021). Assessing school-based policy actions for COVID-19: An agent-based analysis of incremental infection risk. Comput. Biol. Med..

[85] Liu L., Li Y., Nielsen P.V., Wei J., Jensen R.L. (2017). Short-range airborne transmission of expiratory droplets between two people. Indoor Air.

[86] Makris R., Tawackolian K., Lausch K., Kopic C., Kriegel M. (2022). Near-Field Exposure of Pathogen-Laden Respiratory Particles Based on Statistical Evaluation of One Emitting Person Indoors. https://www.researchgate.net/publication/361312085_Near-field_exposure_of_pathogen-laden_respiratory_particles_based_on_statistical_evaluation_of_one_emitting_person_indoors.

[87] Gold J.A.W., Gettings J.R., Kimball A., Franklin R., Rivera G., Morris E., Scott C., Marcet P.L., Hast M., Swanson M. (2021). Clusters of SARS-CoV-2 Infection Among Elementary School Educators and Students in One School District—Georgia, December 2020-January 2021. MMWR Morb. Mortal Wkly. Rep..

[88] Ismail S.A., Saliba V., Lopez Bernal J., Ramsay M.E., Ladhani S.N. (2021). SARS-CoV-2 infection and transmission in educational settings: A prospective, cross-sectional analysis of infection clusters and outbreaks in England. Lancet Infect Dis..

[89] Du C.R., Wang S.C., Yu M.C., Chiu T.F., Wang J.Y., Chuang P.C., Jou R., Chan P.C., Fang C.T. (2020). Effect of ventilation improvement during a tuberculosis outbreak in underventilated university buildings. Indoor Air.

